# Cyclosporine A-related neurotoxicity after haploidentical hematopoietic stem cell transplantation in children with hematopathy

**DOI:** 10.1186/s13052-021-01037-0

**Published:** 2021-04-01

**Authors:** Yong Wang, Yongzhi Zheng, Jingjing Wen, Jinhua Ren, Xiaohong Yuan, Ting Yang, Jianda Hu

**Affiliations:** 1 Department of Pediatric, Fujian Medical University Union Hospital, No. 29 Xinquan Road, Gulou District, Fuzhou City, Fujian Province China; 2 Department of Hematology, Fujian Institute of Hematology, Fujian Provincial Key Laboratory of Hematology, Fujian Medical University Union Hospital, No. 29 Xinquan Road, Gulou District, Fuzhou City, Fujian Province China

**Keywords:** Cyclosporine a, Neurotoxicity syndrome, Hematopoietic stem cell transplantation, Child, Hematopathy

## Abstract

**Background:**

To evaluate cyclosporine A (CSA)-related neurotoxicity after haploidentical hematopoietic stem cell transplantation (HID-HSCT) in children with hematopathy.

**Methods:**

This retrospective case series study included children with hematopathy who underwent HID-HSCT at Fujian Medical University Union Hospital between February 2013 and January 2017.

**Results:**

Fifty-one children (39 males) were included in the study with a median age of 8 (range, 1.1–18) years. Seven patients (13.7%) developed CSA-related neurotoxicity after a median 38 (range, − 3 to 161) days from HID-HSCT. Hypertension (5/7, 71%) was the most common prodrome. Brain magnetic resonance imaging showed posterior reversible encephalopathy syndrome in six patients and atypical abnormalities in one patient. One patient died from grade IV graft-versus-host disease (GvHD) on day + 160, and six patients were alive at the last follow-up. Four patients (71.4%) achieved complete remission, while two patients developed secondary epilepsy and exhibited persistent MRI and electroencephalogram abnormalities at the 5-year follow-up. Hypertension after CSA was more common in patients with CSA-related neurotoxicity than in those without (71% vs. 11%, *P* = 0.002). Five-year overall survival did not differ significantly between patients with CSA-related neurotoxicity (85.7 ± 13.2%) and those without (65.8 ± 7.2%).

**Conclusions:**

The incidence of CSA-related neurotoxicity in children with hematopathy undergoing HID-HSCT is relatively high.

## Background

Hematopoietic stem cell transplantation (HSCT) is the most effective curative therapy for a variety of hematological disorders [1]. Excitingly, the success of haploidentical HSCT (HID-HSCT) has ushered in a new era wherein “everyone has a donor” [2]. The number of children with hematopathy who undergo HID-HSCT has increased dramatically in China, where donor sourcing has been complex because the former one-child policy resulted in sibling donors being a relative rarity [3]. However, the success of HSCT is restricted by graft-versus-host disease (GvHD), a serious complication that affects quality of life and is the main cause of death after transplantation [4, 5]. Prophylaxis against GvHD usually involves the administration of a calcineurin inhibitor, such as cyclosporine A (CSA) or tacrolimus, together with an immunosuppressant, such as low-dose methotrexate or mycophenolate, although alternative agents are also available [6].

CSA is the cornerstone of GvHD prophylaxis. However, neurotoxicity is one of the most common early complications occurring with CSA usage in clinical practice because CSA has a narrow therapeutic index and large inter-individual and intra-individual variability in its pharmacokinetics [7, 8]. Neurotoxicity occurs in 5–11% of patients receiving CSA as GvHD prophylaxis after HSCT [9–13]. The presentation of CSA-related neurotoxicity includes impaired consciousness, seizures, visual disturbance, headache, involuntary movements and paresis [9–14]. Magnetic resonance imaging (MRI) usually shows radiologic features of posterior reversible encephalopathy syndrome (PRES) such as focal regions of symmetric edema affecting the white mater of the parietal and occipital lobes [15]. CSA-related neurotoxicity usually resolves completely with dose reduction or drug withdrawal, but this can have major implications on clinical outcomes, particularly in the face of ongoing GvHD. Furthermore, some CSA-related neurological lesions are irreversible and associated with the later occurrence of epilepsy and persistent abnormalities in the electroencephalogram (EEG) [16]. However, little information is available regarding CSA-related neurotoxicity in children undergoing HID-HSCT.

In this study, we retrospectively examined the data of children who underwent HID-HSCT in order to describe the risk factors and long-term outcomes of CSA-related neurotoxicity. Our aim was to identify factors that potentially could be targeted to prevent post-transplantation CSA-related neurotoxicity or facilitate its early diagnosis and thereby improve survival outcomes and quality of life for patients following HID-HSCT.

## Methods

### Study design and patients

This retrospective case series study included pediatric patients treated with HID-HSCT at the Department of Hematology, Fujian Medical University Union Hospital, Fuzhou, Fujian, China between February 2013 and January 2017. The inclusion criteria were: 1) age ≤ 18 years; 2) received HID-HSCT from a family member who shared one HLA haplotype with the recipient (but differed to varying degrees with regard to the HLA-A, −B and -DR antigens of the unshared HLA haplotype); and 3) received CSA as prophylaxis against GvHD. Patients with neurological disorders before HID-HSCT, including cerebral hemorrhage and central nervous system leukemia, were excluded. The Ethics Committee of Fujian Medical University Union Hospital approved the study (No. 2020ky043) and waived the requirement for informed consent because the analysis was retrospective.

### CSA administration

The FA5-BUCY conditioning regimen and GvHD prophylaxis were administered as described previously by Yang et al. [17]. Briefly, all 51 patients underwent aplasia-inducing salvage therapy consisting of 30 mg/m^2^/day fludarabine and 2 g/m^2^/day Ara-C (cytarabine) for 5 consecutive days from day − 13 to day − 9, followed after 1 day of rest by 3.2 mg/kg/day BU from day − 7 to day − 5 and 1.8 g/m^2^/day CY from day − 4 to day − 3. GvHD prophylaxis consisted of rabbit anti-thymocyte globulin (Thymoglobulin [Genzyme] at 10 mg/kg, 20 patients; ATG-Fresenius® at 40 mg/kg, 23 patients) from day − 4 to day-1, CSA (plasma levels of 100–250 ng/ml; starting from day − 10 intravenously at 1.5 mg/kg every 12 h, followed by oral administration at 5–6 mg/kg/day after completion of the transplantation, and tapering from the second or third month if no signs of GvHD were present), mycophenolate mofetil (5 mg/kg bid, starting from day + 7 and tapered after engraftment), and short-term methotrexate (MTX, 15 mg/m^2^ at day + 1, and 10 mg/m^2^ at day + 3 and + 6). All patients received methylprednisolone for preventing serum sickness associated ATG at 0.8 mg/kg/day from day − 4 to granulocyte implantation. All stem cells were derived from the bone marrow or peripheral blood of the patients’ fathers.

### Monitoring for adverse effects

Monitoring for CSA-related adverse effects consisted of physical examination, blood pressure measurements and routine blood biochemistry tests (including magnesium level), which were performed daily up to day 30 and then 3 times weekly up to day 60. Hypertension was defined according to the 2010 Chinese guidelines for the management of hypertension in children and adolescents (blood pressure above the 75th percentile for age and weight) [18] and was treated with oral calcium channel antagonists. Fluid overload was considered if hypertension or neurological symptoms developed and was treated with intravenous furosemide. Magnesium supplementation was given if plasma magnesium levels fell below 0.8 mmol/L.

Since there are no definitive criteria for CSA-related neurotoxicity, this adverse effect was diagnosed clinically using similar guidelines to those described in previous reports [13, 14, 19]. The diagnosis of CSA-related neurotoxicity was based on the appearance of neurological manifestations during CSA administration (including altered consciousness, seizures, tremors and continuous headache) and the exclusion of other conceivable causes (such as infection, cerebral hemorrhage, metabolic abnormalities, central nervous system leukemia and electrolyte disturbances). If CSA-related neurotoxicity was suspected, CSA was immediately discontinued, and the patient was examined using computed tomography (CT), MRI and electroencephalography. Furthermore, the amelioration of neurological symptoms after the withdrawal of CSA was taken as further evidence in support of a diagnosis of CSA-related neurotoxicity. MRI was performed to exclude other diagnoses and to look for radiological manifestations of PRES (which would support the diagnosis of CSA-related neurotoxicity). In cases with manifestations of epilepsy, electroencephalography was repeated after 24 h and again at 1 month following control of the acute symptoms with anticonvulsants. Electroencephalography was then performed every 3 months for 1 year and annually thereafter if there was no recurrence of seizures or every 3–6 months if recurrence occurred.

### Statistical analysis

For the analysis, the patients were divided into two groups (neurotoxicity group and non-neurotoxicity group) based on whether CSA-related neurotoxicity occurred. Categorical variables are presented as *n* (%) and were compared between groups using Fisher’s exact test or the chi-squared test. Continuous variables were tested for normality, and all the datasets were found not to be normally distributed. Therefore, continuous variables are expressed as median (range) and were compared between groups using the Mann-Whitney U test. Deaths and relapses were considered as competing events, and treatment-related mortality (TRM) was determined using Kaplan-Meier analysis by the log-rank method. The null hypothesis was rejected for *P*-values < 0.05. Statistical analyses were performed using SPSS version 21 (IBM, Armonk, NY, USA).

## Results

### Clinical characteristics of the patients included in the analysis

A total of 51 pediatric patients (39 males, 76.5%) with a median age of 8 (range 1.1–18) years were included in this study. The clinical characteristics of these 51 patients are summarized in Table 1.

**Table 1:** Clinical characteristics of the 51 children who underwent haploidentical hematopoietic stem cell transplantation

Characteristic	Value
Age at HID-HSCT (years), median (range)	8 (1.1–18)
Gender, *n* (%)	
Female	12 (23.5%)
Male	39 (76.5%)
Primary disease, *n* (%)	
Acute lymphoblastic leukemia	14 (27.4%)
Acute myeloid leukemia	25 (49.0%)
Myelodysplastic syndrome-secondary acute myeloid leukemia	4 (7.9%)
Advanced myelodysplastic syndrome	2 (3.9%)
Acquired severe aplastic anemia	6 (11.8%)
^a^Remission status, *n* (%)	
First complete remission (CR1)	24 (55.8%)
Second complete remission (CR2)	5 (11.6%)
Not in remission	14 (32.6%)
Relationship of donor to recipient, *n* (%)	
Parent	49 (96.1%)
Sibling	2 (3.9%)
ABO blood type match between donor and recipient, *n* (%)	
Matched	30 (58.8%)
Mismatched	21 (41.2%)
Donor-recipient gender, *n* (%)	
Male-male	18 (35.3%)
Male-female	9 (17.6%)
Female-female	3 (5.9%)
Female-male	21 (41.2%)
Time to engraftment (days), median (range)	
Neutrophils	12 (10–22)
Thrombocytes	13 (7–35)
Number of CD34+ cells infused (× 10^6^/kg), median (range)	5.33 (2.3–28)
Follow-up time (days), median (range)	405 (44–1432)

^a^Remission status is for 43 children with acute leukemia. HID-HSCT, haploidentical hematopoietic stem cell transplantation

### Clinical characteristics of the patients diagnosed with CSA-related neurotoxicity

Eleven of the 51 children (21.5%) who received HID-HSCT during the study period developed seizure disorders or encephalopathy, but 4 of these 11 children was excluded of CSA-related neurotoxicity due to obvious alternative causes (cerebral hemorrhage in 2 patients, CNS infection in 1 patient and metabolic encephalopathy in 1 patient). Therefore, 7 patients (13.7%) were diagnosed with CSA-related neurotoxicity (neurotoxicity group). The 7 children with CSA-related neurotoxicity included 5 boys and 2 girls with a median age of 7 (range, 4–9) years. The median time to neutrophil and thrombocyte engraftment was 11 (range, 10–19) days and 12 (range, 10–22) days, respectively. The clinical characteristics of the 7 patients with CSA-related neurotoxicity are summarized in Table 2.

**Table 2:** Clinical characteristics of the 7 patients diagnosed with cyclosporine A-related neurotoxicity

Patient number	#1	#2	#3	#4	#5	#6	#7
Gender	M	F	M	M	F	M	M
Age at HID-HSCT (years)	8	7	7	9	8	7	4
Underlying disease	MDS	ALL	AML	MDS-SAML	AML	AML	ALL
Remission status	NR	CR1	CR1	CR1	CR1	CR1	PR
HLA typing	6/10	5/10	7/10	5/10	5/10	5/10	5/10
Donor-recipient gender	F-M	F-F	F-M	F-M	M-F	M-M	M-M
ABO type of donor and recipient	Matched	Mismatched	Mismatched	Mismatched	Matched	Mismatched	Matched
Mononuclear cells (× 10^8^/kg)	6.14	13.1	17.5	17.45	5.6	1.82	16.37
CD34+ cells (×10^6^/kg)	2.3	2.5	6.85	3.17	8.81	10.14	5.33
Neutrophil engraftment time (days)	11	10	11	19	12	16	10
Thrombocyte engraftment time (days)	15	10	11	22	12	17	10

*ALL* Acute lymphoblastic leukemia, *AML* Acute myeloid leukemia, *CR* Complete remission, *F* Female, *HID-HSCT* Haploidentical hematopoietic stem cell transplantation, *M* Male, *MDS-SAML* Myelodysplastic syndrome-secondary acute myeloid leukemia, *NR* No response, *PR* Partial remission

The median number of days from HID-HSCT to neurotoxicity was 38 (range, − 3 to 161) days. The diagnosis of CSA-related neurotoxicity was made during the conditioning stage in 1 patient, at 0–100 days after transplantation in 4 patients, and after day 100 in 2 patients. During CSA dose adjustment, the trough plasma level of CSA ranged from 107.8 ng/mL to 584 ng/mL (the CSA dose was reduced whenever the level exceeded 250 ng/mL). All 7 patients who developed neurotoxicity presented with prodromes 1–2 days before overt neurotoxicity, and the prodromes included hypertension (*n* = 5), transient headache (*n* = 3), vomiting (*n* = 2), fatigue (*n* = 2), dysphoria (*n* = 2) and visual hallucinations (*n* = 1). At the time of overt neurotoxicity, 5 cases were complicated with GvHD, 5 cases with infection, 3 cases with hemorrhagic cystitis, and 5 cases with hypertension. CSA-related neurotoxicity manifested as generalized seizures (*n* = 7), persistent headache (*n* = 3), tremors (*n* = 2), visual disturbance (*n* = 2), psychosis (*n* = 1), aphasia (*n* = 1) and dysphagia (*n* = 1). Plasma levels of CSA were within the desired range in all patients (median, 82.1 ng/mL; range, 57.7–118.3 ng/mL) at the time that neurological symptoms appeared. CSF examinations were performed in 5 patients and returned normal findings in all cases.

### Neuroimaging and electroencephalography

**Fig. 1:**
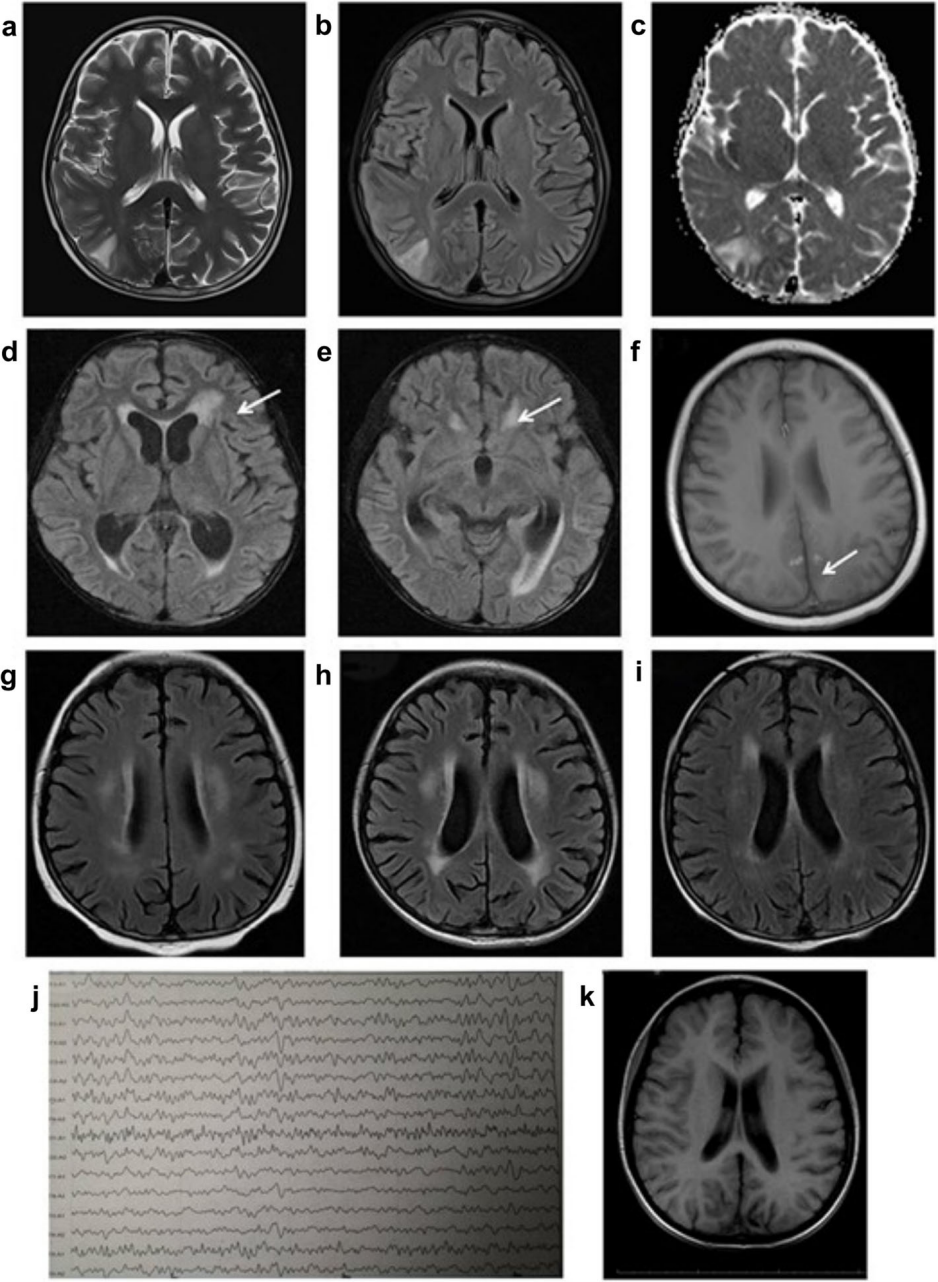
Brain magnetic resonance imaging (MRI) and electroencephalography. **a**, **b**, **c** MRI of the brain of patient #1 showing asymmetrical involvement of the cortex and subcortical white matter with high-intensity signals evident on axial T2-weighted. **a** T2-fluid attenuation inversion recovery (T2-FLAIR). **b** Apparent diffusion coefficient (ADC). **c** Scans. Similar MRI findings were obtained in five other patients with cyclosporine A-related neurotoxicity (patients #2, #3, #4, #5 and #7). **d** and **e.** Axial FLAIR MRI of the brain of patient #6 (obtained 3 days after the onset of symptoms) showing hyperintense signals in the periventricular white matter (white arrow in **d** and **e**) as well as the thalamus (blue arrow in **d**) and hippocampus (blue arrow in **e**). **f** Axial T1-weighted MRI of the brain of patient #4 obtained 3 days after the onset of symptoms. This patient had cerebral hemorrhage associated with posterior reversible encephalopathy syndrome that manifested as a parenchymal hematoma and small hemorrhages < 5 mm in size. The white arrow indicates a microbleed in the occipital lobe. **g**, **h** and **i.** Follow-up MRI of the brain of patient #2. **g** Patchy shadows with high signal intensity were observed in the lateral periventricular white matter 3 days after the onset of symptoms. **h** Hyperintense patchy shadowing in the lateral periventricular white matter was increased at 1 month after onset, despite an improvement in clinical symptoms at this time. **i** Patchy shadowing was less evident at 6 months after symptom onset. **j** and **k.** Electroencephalography and MRI findings in patient #3. MRI showed local cortical atrophy in the left parietal and occipital lobes, and the electroencephalogram demonstrated a spike rhythm originating from the same region of the brain

Neuroimaging and electroencephalography were performed in all patients with CSA-related neurotoxicity. Emergency CT scanning was carried out within 24 h of the appearance of neurological symptoms to exclude intracranial hemorrhage. In patient #4, CT demonstrated a minimal subdural hemorrhagic layer compatible with post-epileptic trauma. MRI was performed within 3 days of the appearance of neurological symptoms after the patient’s vital signs had stabilized (our hospital did not have access to emergency MRI). In 6 of the 7 patients, MRI showed asymmetric involvement of the posterior portions of the cerebral hemispheres, and hyperintense signals were evident on apparent diffusion coefficient (ADC) maps that were consistent with vasogenic edema (Fig. 1a). In the remaining case (patient #6), MRI demonstrated an atypical abnormality that manifested as hyperintense signals in the thalamus and hippocampus predominantly involving the white matter but also the cortex (Fig. 1b). Patient #4 had a complicated cerebral hemorrhage that was associated with PRES (Fig. 1 c). Follow-up MRI examinations were obtained over a period of 2–4 months after the first MRI scan in the 6 children who survived despite developing CSA-related neurotoxicity. Four of these 6 patients exhibited complete resolution of the MRI abnormalities at a median of 2 months (range, 2–8 months) after CSA withdrawal, while 2 cases (patients #2 and #3) had MRI abnormalities that persisted over 3 years (Fig. 1d and e).

It was possible to record an EEG at the time of neurological symptom onset in 6 of the 7 children. The main EEG abnormalities included high-voltage slow-waves (*n* = 5), sharp waves (*n* = 4), spikes (*n* = 3) and spike-wave complexes (*n* = 3). The EEG demonstrated status epilepticus in 3 of the 6 patients and focal delta activity and focal epileptiform discharges in the other 3 patients. Non-epileptiform EEG abnormalities were still detectable in 5 patients (#1, #4, #5, #6 and #7) during the 2–8-week period after the onset of symptoms. Long-term follow-up electroencephalography examinations were performed in 2 cases (patients #2 and #3) with persistent neurological symptoms; the resolution of their EEG abnormalities correlated with their clinical improvement.

### Management of CSA-related neurotoxicity

The treatments administered to the 7 patients with CSA-related neurotoxicity are summarized in Table 3. CSA was withdrawn immediately following the occurrence of neurotoxicity. Tacrolimus alone (*n* = 2) or with mycophenolate mofetil (*n* = 5) was initiated 2 days after CSA washout, with monitoring of the plasma levels. Seizures were treated immediately with intravenous diazepam, and this was followed by anticonvulsive therapy with midazolam. Patients #3 and #4 also required sodium valproate injections to control status epilepticus. Anticonvulsive therapy was tapered and stopped after normalization of the EEG. However, patients #2 and #3 subsequently required anti-epileptic drugs and trihexyphenidyl due to the development of secondary epilepsy (focal epileptiform activity) and tremors. Patient #4 was given anti-psychotic drugs due to the development of psychosis. Additionally, intravenous mannitol was used in 4 patients to treat cerebral edema secondary to seizure recurrence and confusion, vomiting, headache and blurred vision related to intracranial hypertension. Furosemide and captopril were used to control blood pressure and maintain water, electrolyte and acid-base balance. Other forms of supportive care were also provided as necessary.

**Table 3:** Clinical and treatment-related data for the 7 patients with cyclosporine A-related neurotoxicity

Patient number	#1	#2	#3	#4	#5	#6	#7
Day of onset post-HSCT	+ 38	+ 161	+ 24	−3	+ 1	+ 46	+ 137
Prodromes	Transient headache, hypertension	Vomiting, transient headache, hypertension	Dysphoria, hypertension	Fatigue, hypertension, visual hallucination	Transient headache, vomiting	Dysphoria, hypertension	Fatigue
Acute symptoms	Persistent headache, seizures	Status epilepticus, tremors, dysphagia, persistent headache	Status epilepticus, aphasia, tremors	Status epilepticus, visual disturbance, psychosis	Seizures, visual disturbance, persistent headache	Status epilepticus	Seizures
CSA level at time of neurotoxicity (ng/mL)	118.3	110	89.4	68.3	57.7	67.2	82.1
Maximum trough CSA level before CSA-related neurotoxicity (ng/mL)	307	500	135	211	107.8	118	584
Most severe GvHD grade before CSA-related neurotoxicity	Grade II, skin and gut	Grade III, skin and gut	Grade II, skin	No GvHD	No GvHD	Grade III, gut	Grade IV, gut
Immune suppression after CSA-related neurotoxicity	Tacrolimus, MMF, CS, basiliximab	Tacrolimus, MMF, CS, basiliximab	Tacrolimus, MMF, CS, basiliximab	Tacrolimus, MMF	Tacrolimus	Tacrolimus, CS	Tacrolimus, MMF, CS, basiliximab
Acute treatment for neurological symptoms	Diazepam, midazolam,	Diazepam, midazolam, trihexyphenidyl	Diazepam, midazolam, sodium valproate, trihexyphenidyl	Diazepam, midazolam, sodium valproate	Diazepam, midazolam	Diazepam, midazolam	Diazepam, midazolam
Neurological sequelae	None	Secondary epilepsy	Secondary epilepsy	Psychosis	None	–	None
Chronic treatment/duration	Nil	Oxcarbazepine/3 years	Oxcarbazepine/continuous	Risperidone/2 years	None	–	None
Outcome/^a^last follow-up (days post-HSCT)	Alive/+ 2023	Alive/+ 1909	Alive/+ 1818	Alive /+ 1899	Alive/+ 1798	Dead/+ 160	Alive/+ 958
Cause of death/current complications	Symptom-free	Symptom-free	Secondary epilepsy	Symptom-free	Symptom-free	Grade IV GvHD	Symptom-free

*CS* Corticosteroids, *CSA* Cyclosporine A, *GvHD* Graft-versus-host disease, *HSCT* Hematopoietic stem cell transplantation, *MMF* Mycophenolate mofetil. ^a^As of 15 November 2018, except for patient #6 for whom the last follow-up was 29 May 2016

### Outcomes

Death occurred in 1 of the 7 patients with CSA-related neurotoxicity (patient #6), who developed grade IV GvHD and disseminated intravascular coagulation at 5 months post-HSCT and subsequently died from hemorrhagic shock and respiratory failure on day + 160 without neurological symptoms. The remaining 6 patients were alive at the last follow-up. Three of these 6 patients had neurological sequelae, including secondary epilepsy (patients #2 and #3) and psychosis (patient #4). The 2 patients with secondary epilepsy were administered oxcarbazepine, which was successfully withdrawn in patient #2 after 3 years. However, patient #3 required continuous treatment with the anti-epileptic agent because drug withdrawal for 3–6 months resulted in EEG abnormalities and seizure recurrence characterized by sensory disturbances. Patient #4 was given risperidone as an anti-psychotic agent and did not have symptom recurrence. Five patients were alive and symptom-free at a median follow-up of 61.9 (range, 31.9–67.4) months after HSCT (Table 3).

### Risk factors and prognosis

**Fig. 2:**
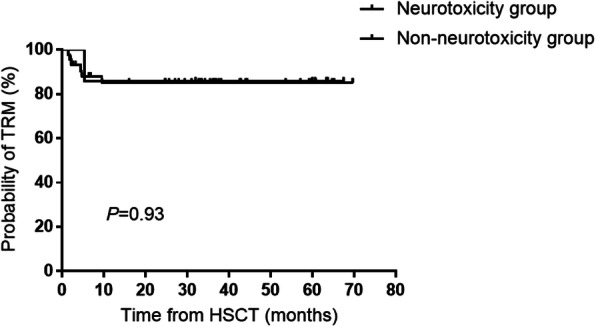
Kaplan-Meier analysis of treatment-related mortality (TRM) in the neurotoxicity and non-neurotoxicity groups. TRM rates were 87.71 and 85.15% in the neurotoxicity (*n* = 7) and non-neurotoxicity (*n* = 44) groups, respectively, with no significant difference between the two groups (*P* = 0.93)

Univariate analysis revealed that the incidence of hypertension during treatment with CSA was significantly higher in the neurotoxicity group than in the non-neurotoxicity group (71% vs. 11%, *P* = 0.002; Table 4). However, there were no significant differences between groups with regard to age at HSCT, gender, underlying diseases, electrolyte imbalance, maximum CSA level, ABO blood type matching between donor and recipient, or gender matching between donor and recipient (Table 4). Treatment-related mortality (TRM) rates were 87.71 and 85.15% in the neurotoxicity (*n* = 7) and non-neurotoxicity (*n* = 44) groups, respectively, with no significant difference between the two groups (*P* = 0.93), as shown in Fig. 2.

**Table 4:** Comparison of clinical characteristics between patients with cyclosporine A-related neurotoxicity and those without

Characteristic	Neurotoxicity	Non-neurotoxicity	***P***
No. of patients	7	44	
Male gender, *n* (%)	5 (72%)	34 (78%)	0.662
Age at HID-HSCT (years), median (range)	7 (1.6)	8 (4.4)	0.212
Underlying disease, *n* (%)			0.221
Acute leukemia	6 (86%)	38 (87%)	
Myelodysplastic syndrome	1 (14%)	1 (2%)	
Aplastic anemia	0 (0%)	5 (11%)	
State of underlying disease at HSCT, n (%)			0.685
Complete remission	5 (72%)	25 (57%)	
Not in remission	2 (28%)	19 (43%)	
Hypertension after CSA, *n* (%)	5 (71%)	5 (11%)	0.002
Electrolyte imbalance, *n* (%)			
Hyponatremia	3 (43%)	7 (16%)	0.095
Hypokalemia	6 (86%)	33 (75%)	0.223
Hypocalcemia	5 (71%)	18 (41%)	0.221
Hypomagnesemia	2 (29%)	10 (23%)	0.662
Maximum trough CSA level > 250 ng/mL, *n* (%)	3 (43%)	12 (27%)	0.406
ABO blood type of donor and recipient, *n* (%)			0.427
Matched	3 (43%)	27 (61%)	
Mismatched	4 (57%)	17 (39%)	
Gender of donor and recipient, *n* (%)			0.923
Matched	3 (43%)	18 (41%)	
Mismatched	4 (57%)	26 (59%)	
Acute GvHD, *n* (%)			0.300
Yes	5 (72%)	38 (86%)	
No	2 (28%)	6 (14%)	

*CSA* Cyclosporine A, *HID-HSCT* Haploidentical hematopoietic stem cell transplantation, *SD* Standard deviation, *GvHD* Graft-versus-host disease

## Discussion

A notable finding of this study was that 13.7% of children who underwent HID-HSCT for hematopathy developed CSA-related neurotoxicity after a median 38 days. Furthermore, 6 of the 7 patients with CSA-related neurotoxicity had MRI features consistent with PRES, although 1 patient exhibited atypical abnormalities. Hypertension was the most common prodrome, occurring in 71% of those who developed CSA-related neurotoxicity. Importantly, hypertension during prophylaxis with CSA was more common in patients with CSA-related neurotoxicity than in those without neurotoxicity (71% vs. 11%). Our findings indicate that CSA-related neurotoxicity is not uncommon in children with hematopathy undergoing HID-HSCT. Furthermore, blood pressure should be carefully evaluated during the early post-transplantation period, since hypertension was the most common prodrome and the only factor in our analysis that differed significantly between patients with CSA-related neurotoxicity and those without.

In this case series, the incidence of CSA-related neurotoxicity was 13.7% in children with hematological malignancies or aplastic anemia treated with HID-HSCT. This incidence was higher than 4.7% reported by a previous Italian multi-center retrospective study [20]. This might be based on the following reasons. On the one hand, the patients in the Italian study mostly had nonmalignant hematological diseases, and previous studies have suggested that the incidence of CSA-related neurotoxicity after HSCT is higher in patients with malignant disorders (9–28.8%) compared with those with nonmalignant disorders (4–11%) [14, 21]. On the other hand, transplantation was mostly HLA-matched allo-HSCT and autologous HSCT in Zama et al. [20], while HLA-unmatched HSCT is considered a risk factor for PRES [22]. In addition, the incidence of CSA-related neurotoxicity may be associated with donor type. For example, Faraci et al. reported that the rate of neurological complications varied with the source of stem cells (27.1% for cells from unrelated donors, 13.9% for cells from related donors, and 2.3% for autologous cells) [11]. Similarly, Koh et al. described neurotoxicity rates of 18.0% for unrelated donors, 11.1% for mismatched related donors and 3.3% for matched related donors [10]. Moreover, Elgarten et al. reported no cases of calcineurin inhibitor-related neurotoxicity in pediatric patients with malignant and nonmalignant diseases who underwent HSCT with matched sibling donors [23]. Since Zimmer et al. also found that the frequency of CSA-related neurotoxicity increased with greater HLA disparity between donor and recipient [24], it is likely that our rate of 13.7% would be higher than that for children who receive HSCT from HLA-matched siblings.

Several factors may be associated with a higher incidence of CSA-related neurotoxicity in pediatric patients who undergo HID-HSCT. First, patients with GvHD above grade II may be at an increased risk of neurotoxicity [25]. Second, CSA-related neurotoxicity is more likely to occur in patients undergoing HSCT from unrelated or unmatched donors [26], which in part may be due to differences in GvHD incidence and severity. Third, the use of intravenous busulfan in the preparative regimen can induce seizures, and this may play a role in the subsequent appearance of CSA-related neurotoxicity [14]. Fourth, younger children (< 6 years of age) tend to suffer more severe seizures in the acute stage than older patients and are relatively more likely to develop epilepsy and neurotoxicity disorders in the future [19]. The vertebrobasilar circulation in children has reduced adrenergic innervation [27], and is less tolerant to changes in arterial blood pressure [28]. Therefore, young children may be particularly susceptible to CSA-related neurotoxicity. Fifth, there is evidence that hypertension may be associated with CSA-related neurotoxicity. For example, hypertension has been reported as a prodrome to CSA-related neurotoxicity in 83% [14] and 46% [29] of patients, which is broadly comparable to our finding that 71% of patients exhibited hypertension as a prodrome. Furthermore, hypertension after CSA treatment was the only factor in our analysis associated with an increased risk of CSA-related neurotoxicity.

Neuroimaging now plays a central role in the diagnosis and long-term follow-up of CSA-related neurotoxicity. CT is typically the first tool used to investigate patients with acute neurological disorders, but this imaging technique identifies lesions in only about 50% of PRES cases [30]. Since children receiving transplants can present with a wide spectrum of acute neurologic complications, MRI-based evaluation is essential for the accurate diagnosis of PRES. DWI and ADC maps can help to distinguish vasogenic edema from cytotoxic edema [31]. Vasogenic edema typically presents as hyperintensity on ADC and isointensity or hypointensity on DWI, while cytotoxic edema usually manifests as hypointensity on ADC and hyperintensity on DWI [32]. Although CSA-related neurotoxic events are typically thought to be confined to fluid extravasation, more severe endothelial damage has been reported, including erythrocyte extravasation with parenchymal hemorrhage [33]. Cerebral hemorrhage is associated with PRES in 5–19% of cases [34], which might explain the focal hemorrhages observed in the MRI scans of patient #4 in our study.

The clinical and imaging manifestations of PRES are reversible in the majority of patients (70%) provided CSA is promptly reduced in dosage or withdrawn, but a delay in the diagnosis and treatment of PRES can result in irreversible neurological damage [33]. Karia et al. reported a strong association between MRI severity and clinical outcome [35]. Furthermore, children with persistent EEG or imaging abnormalities have been reported to be at higher risk of seizure recurrence following CSA-related neurotoxicity [36]. Cerebral hyperperfusion due to hypertension and aggravation of vascular endothelial injury may be important mechanisms by which CSA increases the permeability of the blood-brain barrier [37]. Irrespective of the underlying pathology, MRI and electroencephalography can be extremely useful for the detection of lesions due to CSA-related neurotoxicity and the long-term follow-up of patients with persistent abnormalities. In the present study, three patients with persistent EEG or imaging abnormalities had neurological sequelae. However, two patients subsequently recovered, and the symptoms in the other patient were controlled after standardized treatment.

In the case of severe hypertension during the acute phase of neurotoxicity, arterial blood pressure should be reduced by ∼25% within the first hour (after the exclusion of cerebral infarction) and then more slowly [38]. CSA should be discontinued following the occurrence of neurotoxicity, and tacrolimus is the most commonly used alternative agent in the event of CSA-related neurotoxicity [13, 34, 39]. Although tacrolimus is also associated with neurotoxicity [13, 39], it did not produce neurological adverse effects in this series of patients. Anti-convulsive therapy should be administered as early as possible to control ongoing seizures. Prolonged prophylactic treatment with anti-epileptic drugs is unnecessary in patients with occasional or provoked seizures due to CSA-related neurotoxicity, but such drugs should be considered in cases with later development of secondary epilepsy [34]. In the present study, 1 of the 7 patients with CSA-related neurotoxicity (14%) required long-term anti-convulsive therapy.

The 5-year OS rate for the pediatric patients who developed CSA-related neurotoxicity after HID-HSCT was not significantly different to that for the patients who did not exhibit neurotoxicity. This is a promising finding, as it suggests that appropriate detection and intervention can ameliorate the long-term consequences of these relatively common adverse events.

This study has some limitations. First, this was a retrospective analysis, so the results are prone to information bias and selection bias. In addition, the rate of hypertension could be underestimated in control patients. Second, this was a single-center study, so the generalizability of the findings is not known. Third, the sample size was small, so the study may have been underpowered to detect some real differences between groups. Fourth, unknown confounding factors may have influenced the analysis. Fifth, CSA-related neurotoxicity was diagnosed on the basis of clinical and radiological findings, but it is possible that some mild cases may have been missed, leading to an underestimation of the incidence.

## Conclusion

There is a relatively high incidence of CSA-related neurotoxicity in children with hematological disorders undergoing HID-HSCT. Awareness of the clinical features of CSA-related neurotoxicity (particularly hypertension) can facilitate rapid diagnosis and timely implementation of appropriate interventions. Moreover, patients with persistent EEG or imaging abnormalities can benefit from MRI and EEG examinations as part of their long-term follow-up program.

## Data Availability

The datasets used and/or analyzed during the current study are available from the corresponding author on reasonable request.

## Abbreviations

ADC: Apparent diffusion coefficient
CSA: Cyclosporine A
CT: Computed tomography
DWI: Diffusion weighted imaging
GvHD: Graft-versus-host disease
EEG: Electroencephalogram
HID-HSCT: Haploidentical hematopoietic stem cell transplantation
MRI: Magnetic resonance imaging
OS: Overall survival
PRES: Posterior reversible encephalopathy syndrome
